# Microbial Responses and Metabolic Mechanisms During Anaerobic Degradation of N,N-Dimethylformamide by Co-Cultured Sludge

**DOI:** 10.3390/microorganisms14061172

**Published:** 2026-05-22

**Authors:** Jianrong Liu, Yingying Song, Hongruo Ma, Chunlan Mao, Zuoyan Chen

**Affiliations:** 1 Gansu Natural Energy Institute, Lanzhou 730000, China; 2 Gansu Analysis and Research Center, Lanzhou 730000, China; 3 State Key Laboratory of Ecological Safety and Sustainable Development in Arid Lands, Lanzhou Eco-Agriculture Experimental Research Station, Northwest Institute of Eco-Environment and Resources, Chinese Academy of Sciences, Lanzhou 730000, China

**Keywords:** N,N-dimethylformamide, anaerobic co-cultured sludge, microbial community, functional genes, methylotrophic methanogenesis, C1 carbon flux

## Abstract

Anaerobic biodegradation is the most affordable method for the degradation of N,N-dimethylformamide. However, the degradation efficiency depends on the concentration. To elucidate the responses of microbial community to N,N-dimethylformamide load, microbial diversity, composition and functional changes at different concentrations of 100, 2000, and 3500 mg/L were analyzed. Results showed that as the N,N-dimethylformamide influent concentration increased from 100 to 2000 mg/L, the removal rate stabilized at 90%, whereas it decreased to ~75% at concentrations over 2000 mg/L. Microbial community diversity increased, and specialists were enriched at 3500 mg/L. Patescibacteria (42.88% and 42.90%), Bacillota (18.52% and 18.54%), and Pseudomonadota (7.13% and 7.09%) were the dominant phyla at 100 mg/L and 2000 mg/L, respectively, and Patescibacteria (16.88%) and Pseudomonadota (15.34%) were the dominant phyla at 3500 mg/L. Methylotrophic methanogeneic (*Methanolobus* and *Methanomassiliicoccus*) and syntrophic electron-donating bacteria (*Clostridium* and *Trichococcus*) were significantly enriched. DMF-degrading genes (*fdh*, *rfA*/*nrfH*, and ATPase) and methylotrophic methanogenesis genes (*mcr*, *mta*, and *mtm*) were significantly upregulated. Therefore, the degradation of N,N-dimethylformamide was characterized by a parallel carbon flux distribution, “methylamine-driven methanogenesis + further oxidation/integration of single-carbon intermediates”, and the nitrogen flux tended to enter a reductive nitrogen cycle characterized by retention and reuse.

## 1. Introduction

N,N-dimethylformamide (DMF) is a polar organic solvent with strong dissolving ability and high chemical stability. It is widely used in industries such as pesticide production, leather manufacturing, and pharmaceutical synthesis [1]. However, the rapid development of related industries has resulted in continuous discharge of industrial wastewater containing DMF. In some industrial effluents, DMF concentrations reached 10,000 mg/L [2]. DMF has high thermal stability but low biodegradability. These drawbacks make it difficult to remove effectively through natural degradation or thermal decomposition once it enters the environment. Consequently, this leads to long-term persistence and accumulation, ultimately generating a continuous threat to ecological environmental safety. Furthermore, DMF exhibits biotoxicity, which can cause significant damage to the liver, kidneys, and immune system, and harm human health through skin contact, inhalation, and ingestion [3,4]. Therefore, the development of green, efficient technologies for treating DMF-containing wastewater is an urgent issue in environmental remediation.

Current treatment methods for DMF organic wastewater include physical methods (extraction [5], adsorption [6], and distillation [7]), chemical methods (Fenton reagent, supercritical water oxidation, and discharge plasma technology [8]), and biological methods. Compared with physical and chemical methods, biological methods offer advantages such as lower operating costs, environmental friendliness, and minimal secondary pollution. Therefore, biological methods are among the most promising technologies for treating of industrial organic wastewater [9]. Under aerobic conditions, the biodegradation of DMF proceeds via two major metabolic pathways [10]. The first is the hydrolytic pathway, in which DMF is converted into dimethylamine (DMA) and formic acid (HCOOH) under the catalysis of N,N-dimethylformamidase (DMFase), and can be further transformed into monomethylamine (MMA). The catalytic role of this key enzyme has been extensively documented in previous studies [11]. The second is the sequential oxidative demethylation pathway, in which DMF is stepwise demethylated to form N-methylformamide (NMF), formaldehyde (HCHO), and formamide (FA) in sequence [12]. Given the well-established degradation pathways, aerobic biological treatment has been commonly adopted for the treatment of low-concentration DMF wastewater.

In contrast, anaerobic digestion (AD) presents distinct merits such as low energy consumption, reduced sludge yield, and excellent organic loading tolerance, and is recognized as a highly promising advanced strategy for high-concentration DMF wastewater treatment. In previous investigations on anaerobic DMF degradation, the degradation of 2000 mg/L DMF and its methylamine-type intermediates has been realized through the synergistic action of DMF hydrolytic bacteria and methylotrophic methanogens [13]. These intermediates are further converted into methane and carbon dioxide by methylotrophic methanogenic archaea, and the entire process relies on the syntrophic cooperation of hydrolytic and fermentative bacteria, syntrophic metabolizing bacteria, and methanogenic archaea [14]. Nevertheless, stable anaerobic degradation of DMF at higher concentrations has not been reported in existing studies. In particular, the microbial tolerance and adaptive mechanisms as well as the distribution patterns of carbon and nitrogen fluxes under high-concentration stress remain largely unknown. In biological treatment systems, the microbial community serves as the core driver for pollutant degradation and stable system operation, and its community structure, diversity, and functional expression are highly sensitive to variations in environmental conditions and substrate loading. Consequently, in-depth illumination of the changes in microbial community and functional response characteristics in anaerobic treatment systems provides an essential scientific basis for uncovering the mechanisms underlying system stability, achieving precise process regulation, and improving DMF degradation efficiency.

Based on our preliminary study, DMF-degrading sludge and methanogenic sludge were co-cultured in an up-flow anaerobic sludge blanket (UASB) reactor and acclimated by gradually increasing the DMF concentration (from 100 to 3500 mg/L) for 60 days. It was found that DMF could be removed efficiently. However, the microbial changes at different DMF concentrations and its metabolic functions remained unclear. Therefore, the present study aimed to further investigate the microbial responses to DMF loads and its metabolic mechanisms in the co-cultured anaerobic system. Accordingly, sludge samples were collected at three representative DMF concentrations: 100 mg/L (low-concentration background level), 2000 mg/L (critical loading for efficient and stable degradation documented in previous studies [14]), and 3500 mg/L (high-toxicity stress loading). Then, changes in community composition and diversity of the three concentrations were analyzed using 16S rRNA high-throughput sequencing, and functional genes involved in DMF degradation of DMF3 and potential degradation pathways were identified using metagenomic sequencing. The DMF removal rates and intermediates were also analyzed to assess the DMF degrading efficiency. These results provide a micro-ecological regulation strategy for optimizing DMF anaerobic treatment processes and enriching the microbial ecological theory of organic pollutant biodegradation.

## 2. Materials and Methods

### 2.1. Experimental Design and Sample Collection

In this study, a UASB reactor with an effective volume of 12 L was employed as the core treatment system. The reactor was operated under mesophilic anaerobic conditions at a precisely controlled temperature of 35 ± 1 °C, with a hydraulic retention time (HRT) of 72 h and the pH value stably maintained in the range of 7.0–7.5. The seed sludge was prepared by mixing anaerobic digested sludge (collected from the anaerobic tank of an industrial wastewater treatment plant in Lanzhou, Gansu Province, China) with methanogenic anaerobic sludge at a volume ratio of 2:1; procedures were as previously described [15]. The resulting co-cultured sludge had a total solids concentration of 15.53 mg/L and an organic matter content of 22.75%. Synthetic DMF-containing wastewater was used as the reactor influent. The initial DMF concentration was set at 100 mg/L with a corresponding initial chemical oxygen demand (COD) concentration of 250 mg/L. The mass ratio of glucose, sodium acetate (supplementary carbon sources) and DMF was controlled at 1:1.5:1 [16]. Considering HRT, and to ensure the metabolic stability of the anaerobic system, influent and effluent samples were collected every 5 days for the determination of key physicochemical parameters. Once the effluent DMF and COD concentrations remained stable for three consecutive sampling events, the concentrations of DMF and supplementary carbon sources were increased by approximately 20%. Each concentration gradient was operated for a minimum of 15 days to ensure system acclimation. After 20 successive operation cycles with a total duration of 500 days, the influent DMF concentration was gradually elevated to 3500 mg/L, and the influent COD concentration reached 9000 mg/L. At the end of the stable operational periods under influent DMF concentrations of 100 mg/L, 2000 mg/L and 3500 mg/L (denoted as DMF1, DMF2, and DMF3 respectively), 50 mL mixed liquor sludge samples were collected respectively for 16S rRNA gene high-throughput sequencing analysis. Additionally, anaerobic sludge samples obtained under the 3500 mg/L DMF condition were subjected to metagenomic sequencing to explore functional gene profiles. All sludge samples were immediately centrifuged at 8000 r/min for 10 min at 4 °C. After discarding the supernatant, the precipitated sludge pellets were flash-frozen and stored at −80 °C until subsequent physicochemical characterization and microbial sequencing analysis.

### 2.2. Physicochemical Index Determination

The concentrations of DMF and its intermediates, DMA and MMA, were determined using high-performance liquid chromatography (LC-2030, Shimadzu, Kyoto, Japan) with a C18 reverse-phase column (250 mm × 4.6 mm, 5 μm). The mobile phase was methanol:water = 40:60 (*v*/*v*) at a flow rate of 1.0 mL/min; the detection wavelength was 205 nm, and the column temperature was 30 °C. COD was determined using the dichromate spectrophotometric method. Ammonia nitrogen (NH_3_-N) was determined using Nessler’s reagent spectrophotometry. Total nitrogen (TN) was measured using alkaline potassium persulfate digestion UV spectrophotometry. pH was measured using a pH meter (PHSJ-6L, Yifen, Shanghai, China). Gas production was recorded daily using the water displacement method, and methane content was determined using gas chromatography (GC-2014, Shimadzu, Japan). The volumetric biogas production rate (VBPR) and volumetric loading rate (VLR) were calculated as kg COD/m^3^/(m^3^·d) and L·L^−1^·d^−1^, respectively.

### 2.3. DNA Extraction and Sequencing

All sludge samples were analyzed by Beijing Novogene Bioinformatics Technology Co., Ltd. (Beijing, China), which used high-throughput sequencing to detect microbial communities. The bacterial primers were as follows: forward primer: CCTAYGGGRBGCASCAG, reverse primer: GGACTACNNGGGTATCTAAT. DNA extraction and 16S rDNA amplification, sequencing and data analysis were performed using the Novomagic cloud platform: https://magic-plus.novogene.com (accessed on 1 December 2025). The raw data obtained from sequencing were spliced and filtered to obtain clean data. Based on the clean data, denoising was performed using DADA2 (v.1.1.456) [17] to obtain the final feature sequences (Amplicon Sequence Variants, ASVs, accessed on 1 December 2025). ASVs were clustered with 97% identity. DNA extraction, metagenomic amplification, and sequencing were performed at Majorbio Bio-Pharm Technology Co., Ltd. (Shanghai, China). We used Majorbio Cloud: https://cloud.majorbio.com (accessed on 20 December 2025) for data analysis.

### 2.4. Data Analysis

Mothur software (v.1.30.1) was used to measure microbial diversity indices. PICRUSt2 was used to analyze the microbial function. Diamond v2.0.13 was used to analyze different genes. Origin 2018 was used to analyze the Spearman correlation coefficient analysis and redundancy analysis (RDA) between the microbial community and the physicochemical properties of DMF wastewater.

## 3. Results

### 3.1. DMF Degrading Efficiency

**Figure 1:**
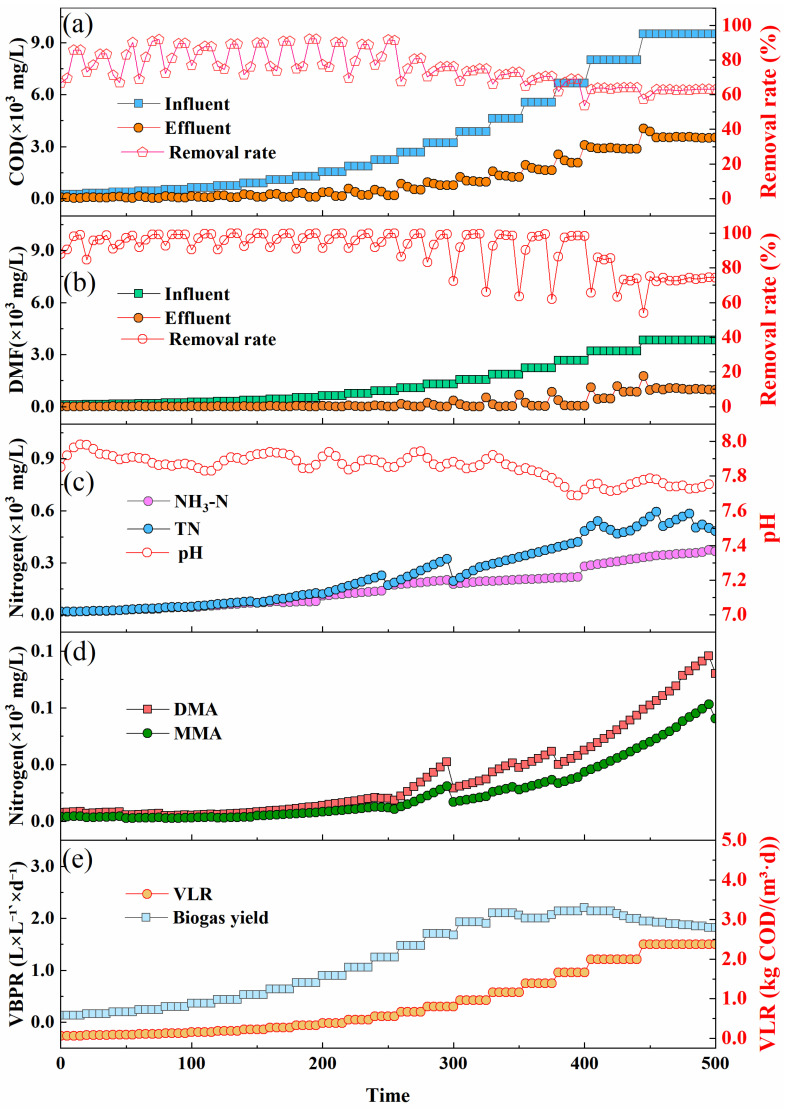
Dynamic characteristics of DMF removal, nitrogen transformation, and intermediate production during acclimation of the UASB reactor. (**a**) COD removal performance; (**b**) DMF degradation performance; (**c**) effluent nitrogen concentration (mg/L) and pH variation; (**d**) production of dimethylamine and monomethylamine (mg/L); (**e**) biogas production performance. COD, chemical oxygen demand; DMF, N,N-dimethylformamide; TN, total nitrogen; NH_3_-N, ammonia nitrogen; DMA, dimethylamine; MMA, monomethylamine; VLR, volumetric loading rate.

The treatment performance of the UASB reactor for synthetic DMF wastewater during acclimation was investigated by gradually increasing the influent organic load. As shown in Figure 1a,b, the influent COD concentration increased stepwise from 250 to 9000 mg/L, corresponding to an increase in the influent DMF concentration from 100 to 3500 mg/L. The effluent COD and DMF concentrations increased synchronously with the influent loads. However, they gradually decreased and stabilized in the later stages of each load increase cycle, indicating that the reactor microbial community could gradually adapt to the high-concentration substrate environment. The COD removal rate remained above 80% throughout the 500-day operating period, even when the influent COD reached 9000 mg/L. This suggests that the co-metabolism of glucose and sodium acetate in the mixed substrate effectively enhanced the activity of the anaerobic microorganisms, providing sufficient reducing power and electron donors for DMF degradation. The DMF removal rate remained stable above 90% at influent concentration ≤2000 mg/L, demonstrating the efficient mineralization capacity of the acclimated functional microorganisms for DMF. The similar result was found that at low OLR of 1.63–4.22 g COD L^−1^ d^−1^, removal rate of 2000 mg L^−1^ DMF exceeded 96% [14]. But the present OLR (0.25–9.0 g COD L^−1^ d^−1^) was much higher than this. However, when the concentration exceeded 2000 mg/L, the removal rate decreased to approximately 75%, indicating that excessive DMF loading inhibited the microorganisms owing to the biotoxicity of DMF and its degradation intermediates. It has been reported that the acute toxicity of sludge was positively correlated to the influent DMF concentration. When DMF concentration ranged from 40 to 200 mg L^−1^, the toxicity increased from 25 to 45% which was primarily contributed by the biodegradation of DMF [18].

A typical pathway for anaerobic DMF degradation is the coupling of demethylation and deamination, in which the pattern of nitrogen release reflects the degradation level. As shown in Figure 1c, the effluent TN concentration gradually increased from 0 to 600 mg/L, as the influent DMF load increased. NH_3_-N accounted for the majority of TN. However, its concentration remained consistently lower than TN, indicating that the amide nitrogen in DMF molecules was primarily released as NH_3_-N, with a small amount of organic nitrogen (e.g., intermediate amines) remaining unmineralized. The system pH remained stable between 7.0 and 7.6, without significant fluctuations. This indicated a well-established acid–base buffering system during anaerobic digestion and no risk of failure due to volatile fatty acid accumulation. The neutral-to-slightly alkaline environment met the optimal growth requirements of methanogens, and also facilitated the release and transformation of NH_3_-N during DMF deamination. This provided a solid basis for the subsequent nitrogen removal.

The key intermediates in the anaerobic degradation of DMF were DMA and MMA, and their concentration changes directly reflected the degradation efficiency of DMF. As shown in Figure 1d, the DMA concentration in the effluent remained between 0 and 90 mg/L, and the MMA concentration was consistently lower than that of DMA. Both increased slowly with increasing DMF loading, without significant accumulation. This indicated that the acclimated microbial community efficiently accomplished the stepwise demethylation reaction DMF → DMA → MMA, and the methylamine intermediates were further converted to CH_4_ and CO_2_ by methylotrophic methanogens. Thus, the inhibition of toxicity by the intermediate products was prevented. During the high-DMF-load phase (>2000 mg/L), DMA and MMA concentrations increased slightly, consistent with the decreasing trend in DMF removal efficiency. This indicates that the demethylation reaction rate was limited under a high load, which was the main reason for the decreased DMF removal efficiency. These findings provide direct evidence for optimizing the UASB operating load and enhancing intermediate degradation.

Changes in VBPR and VLR (Figure 1e) further validated the stability of the system. VLR gradually increased from 0 to 3.0 kg COD (m^3^·d), and VBPR increased synchronously, and then stabilized between 2.0 and 3.0 L·L^−1^·d^−1^. The results indicated that, after acclimation, the reactor achieved stable energy recovery under high organic loads. Combined with the high COD/DMF removal rates, stable pH, and low intermediate accumulation, it can be inferred that the methane content in the biogas remained high, thus achieving the synergistic goal of pollutant removal and bioenergy recovery. Based on the above results, the UASB reactor achieved stable, efficient operation at appropriate influent concentrations of DMF ≤ 2000 mg/L and COD ≤ 9000 mg/L via gradient load acclimation. The COD and DMF removal rates exceed 80% and 90%, respectively. Therefore, the study illustrated the feasibility of the anaerobic degradation of DMF, consistent with previous studies [13,14]. The patterns of nitrogen release and pathways of intermediate degradation were clear, providing a reliable technical basis for the anaerobic biological treatment of DMF wastewater.

### 3.2. Characteristics of Microbial Community

#### 3.2.1. Microbial Community Diversity

High-throughput sequencing was performed on anaerobic sludge samples at three DMF concentrations (100, 2000, and 3500 mg/L). A total of 836,329 quality sequences of bacteria were obtained from the 12 samples, with 56,810 to 101,481 sequences per sample and an average of 92,925 sequences per sample. More than 56,000 effective reads with average lengths of 420.94 bp were obtained for each sample. The coverage of all samples ranged from 0.997 to 0.998 (Table 1), indicating sufficient sequence data for identifying the microbial communities (showed in the rarefaction curves in Figure S1). This indicated that the sequencing depth adequately covered the species diversity and reliably reflected the true microbial community structure of the anaerobic sludge.

**Figure 2:**
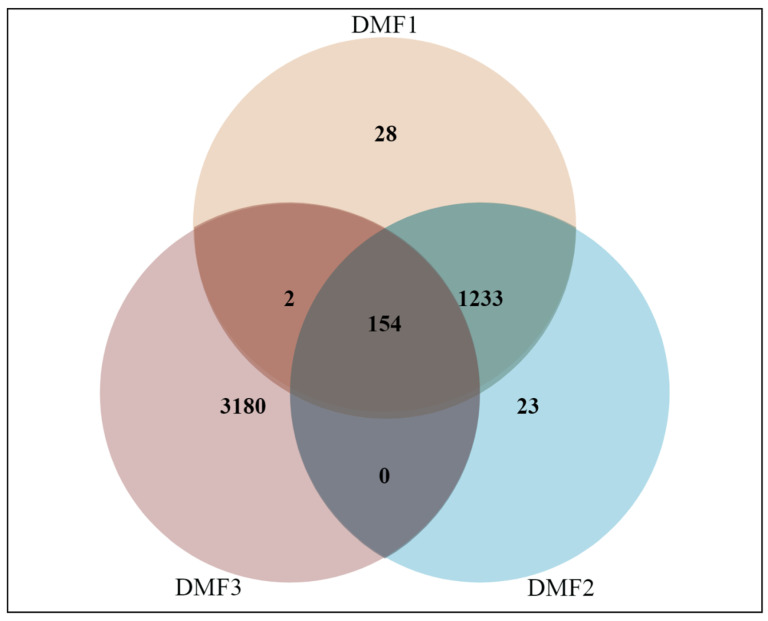
Composition of microbial community at the ASV level in anaerobic sludge under different DMF concentrations.

4680 ASVs belonging to 4637 phyla, 4623 classes, 4575 orders, 4501 families, 4120 genera, and 2905 species were obtained. The Venn diagram analysis of all samples at the three DMF concentrations (Figure 2) shows that there were 154 shared ASVs, accounting for 3.17% of the total ASVs. There were 28, 23, and 3180 unique ASVs for DMF1, DMF2, and DMF3, respectively, accounting for 1.98%, 1.63%, and 95.32% of the total ASVs in each group, respectively. The results indicated that as the DMF concentration increased from 100 to 2000 mg/L, the microbial community composition remained largely unchanged. However, when the DMF concentration was increased to 3500 mg/L, the number of specialists increased significantly. Therefore, it was reconstructed, and some degrading microorganisms with high tolerance were enriched.

Microbial diversity assesses the diversity and richness of microorganisms in samples. Statistical analysis of the diversity indices for the anaerobic sludge samples at the three different DMF concentrations (Table 1) showed that when the DMF concentration increased from 2000 mg/L, the Chao1, Shannon, and Simpson values showed no significant change (*p* > 0.05). When the concentration increased to 3500 mg/L, the values were higher than those at 100 and 2000 mg/L, indicating the highest microbial richness and diversity. This suggested that high DMF concentration increased the richness and diversity of microbial communities in the co-cultured sludge.

#### 3.2.2. Characteristics of Microbial Community Structure

**Figure 3:**
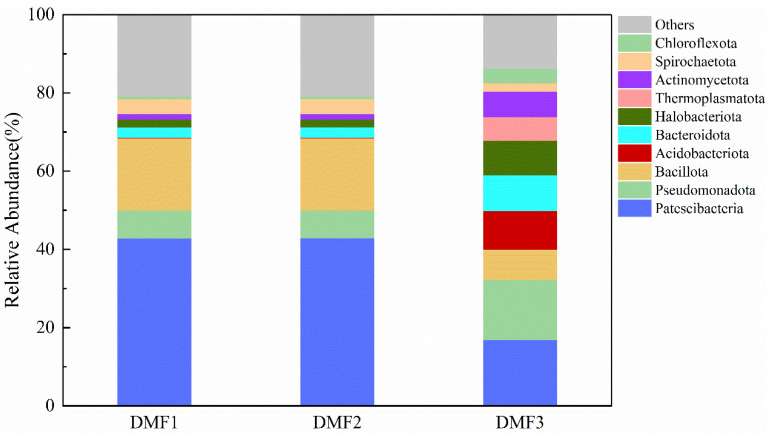
Phylum-level composition of anaerobic sludge microbial communities under different DMF concentrations.

At the phylum level (Figure 3), the dominant phyla at the three DMF concentrations were similar, including Patescibacteria, Pseudomonadota, Bacillota, Acidobacteriota, Bacteroidota, Halobacteriota, Thermoplasmatota, Actinomycetota, Spirochaetota, and Chloroflexota, accounting for over 79.15% of the total microbial abundance. Patescibacteria, Bacillota, and Pseudomonadota were predominant at DMF concentrations of 100 and 2000 mg/L with relative abundances of 42.88% and 42.90%, 18.52% and 18.54%, and 7.13% and 7.09%, respectively. Patescibacteria are a single phylum discovered through metagenomics in recent decades which may constitute over 15% of bacterial diversity [19]. Their epibiotic lifestyle may enable them to provide metabolic functions to their host [19]. Therefore, DMF degradation might be carried out through complementary biochemical functions of Patescibacteria supporting host metabolism and improving adaptations. This is quite different from previous studies, in which Proteobacteria, Firmicutes, and Actinobacteria were dominant [20,21,22]. When the concentration increased to 3500 mg/L, almost all abundances increased except Bacillota and Patescibacteria. This indicated the obvious toxicity adaptation and tolerance enhancement. Although Patescibacteria was the dominant phylum with a relative abundance of 16.88%, it was 26% lower than at the previous two concentrations. The second most dominant phylum was Pseudomonadota, with a relative abundance of 15.34%. It increased by approximately 8.2% compared to the 100 mg/L and 2000 mg/L concentrations. Similarly, Acidobacteriota (9.84%) and Bacteroidota (9.16%) showed increases of 9.7% and 6.5%, respectively, compared to those at 100 and 2000 mg/L. Additionally, the relative abundances of Halobacteriota, Thermoplasmatota, and Actinomycetota increased. Conversely, the relative abundances of Bacillota and low-abundance microbiota (others) decreased by 10.7% and 7.0%, respectively. Therefore, the microbial community of the anaerobic degradation system was significantly restructured, particularly with increases in Actinomycetota, Bacteroidota, Halobacteriota, and Thermoplasmatota, which could be the main functional bacteria with high toxic tolerance to DMF. The results indicated that a high concentration (>2000 mg/L) of DMF exerted significant directional selection on the microbial community of the anaerobic system and gradually enriched the functional groups capable of adapting to organic solvent stress and participating in the metabolism of DMF and its intermediates, ultimately forming a core microbial community with better adaptability to high DMF loads.

Analysis at the genus level revealed a significant succession in the microbial communities, which was closely related to the anaerobic degradation of DMF in the co-cultured sludge as the DMF concentration increased. The genera potentially contributing to DMF degradation at the three DMF concentrations are summarized in Table 2. *Methanolobus*, *Methanomassiliicoccus*, and *unclassified_Methanomassiliicoccaceae* were significantly enriched at a DMF concentration of 3500 mg/L, indicating that under a high load, the terminal metabolism of the system favors methylotrophic methanogenesis using methylamines/methanol as substrates. *Methanomassiliicoccus* and *Methanolobus* as methanotrophic methane-producing archaea were main functional microorganisms contributing to DMF anaerobic degradation [23]. Genera such as *Desulfovibrio*, *Pseudomonas*, and *Hyphomicrobium*, although presented at lower abundances, could be the main hydrolytic bacteria that played key roles in the initial hydrolysis and/or the early transformation stage of DMF. *Pseudomonas* and *Hyphomicrobium* have been proved to be degraders in DMF [23,24]. *Hyphomicrobium* was regarded as a typical methylotrophic bacterium capable of denitrifying. Meanwhile, *Clostridium* and *Trichococcus*, which play an important role in synergistic degradation with degraders [25,26], were the key syntrophic electron-donating bacteria and/or fermentative support bacteria, providing electrons and intermediate metabolites for methanogenesis. Therefore, it could be concluded that in the co-cultured anaerobic system, the degradation of DMF was carried out via a complex microbial network composed of “initial transforming bacteria–methylotrophic methanogenic archaea–syntrophic/co-metabolic bacteria.”

### 3.3. Biological Drivers of DMF Degrading in Genetic Levels

#### 3.3.1. Microbial Growth and Reproduction

**Figure 4:**
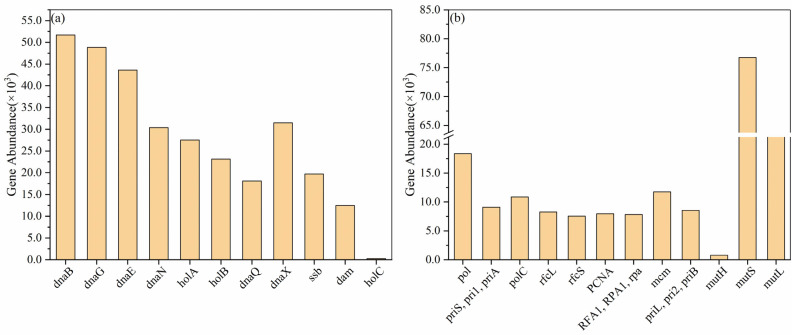
Abundance of DNA replication and mismatch repair genes in bacteria (**a**) and archaea (**b**) in the anaerobic system.

Functional gene analysis indicated that under high DMF stress (3500 mg/L), both bacteria and archaea in the anaerobic system possessed relatively complete DNA replication systems (Figure 4). Bacterial replication genes (e.g., *dnaB*, *dnaG*, and *dnaE*) were upregulated, suggesting a strong reproductive capacity (Figure 4a). Similarly, archaeal genes such as *pol*, *mcm*, and *PCNA* were upregulated, indicating stable replication capability. Furthermore, bacterial DNA mismatch repair genes such as *dam*, *dnaQ*, *dnaX*, *ssb*, and *holC* were highly abundant. It showed that the DMF intermediates exerted biotoxicity on microbial cells. For archaea (Figure 4b), the abundance of mismatch repair genes was higher than that of replication genes, indicating the same biotoxicity of intermediates, lower tolerance, and greater sensitivity, thereby limiting further metabolism of the degraded substrates. The abundance of archaeal mismatch repair genes *mutS* and *mutL* was higher than that of *mutH*. It indicated that a potential mismatch repair pathway, depending on the MutS-MutL type, is required to maintain genomic stability, thus ensuring the continuous operation of terminal methanogenic functions in the anaerobic system.

#### 3.3.2. Functional Genes in Carbon Flow Pathways

**Figure 5:**
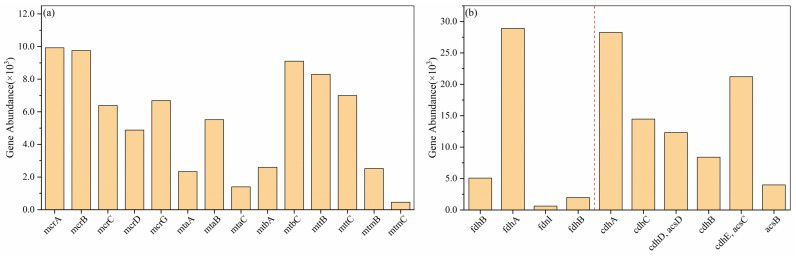
Abundance of genes related to demethylation (**a**) and complete mineralization to CO_2_ (**b**) in the anaerobic system.

In the anaerobic system, methane metabolic genes *mcrA* (M00567, CO_2_ ⇒ methane), *mcrB* (M00357, acetate ⇒ methane), and *mcrG* (M00563, methylamine/dimethylamine/trimethylamine ⇒ methane) obtained higher counts ranging from 6686 to 9924. They were methyl-coenzyme M reductase [EC:2.8.4.1] which could convert DMF intermediates into methane directly. Genes *mcrC* (methyl-coenzyme M reductase subunit C) and *mcrD* (methyl-coenzyme M reductase subunit D) also contributed to methane metabolism with a count of 6380 and 4876, respectively (Figure 5a). Therefore, the methyl-coenzyme M reductase (MCR) complex was relatively complete and abundant, and methanogenesis could be the main terminal metabolic route. This was consistent with the results of gas production. The methylotrophic methanogenesis genes *mta*, *mtb*, *mtt*, and *mtm* were detected, indicating a methanogenesis pathway involving methanol and methylamine substrates. The counts of genes *mtbC*, *mttB*, and *mttC* involved in the methylamine/dimethylamine/trimethylamine ⇒ methane (M00563) route were much higher than those of genes *mtaA*/*C* involved in the methanol ⇒ methane route (M00356), second only to the counts of *mcrA* and *mcrB*. This indicated the methyl transfer pathways for DMA and TMA were dominant in methylotrophic methanogenesis, while those for methanol and MMA were relatively weaker [15,21,27]. These results indicated that methylotrophic methanogenesis was a crucial terminal metabolic pathway in the co-cultured system, and that methylamine intermediates played a dominant role in carbon flux conversion.

Genes encoding formate dehydrogenase (FDH) and those related to C1 carbon flux conversion were also highly abundant (Figure 5b). Among these, *fdhA*, *fdhB*, and *fdnI* encoded different subunits of formate dehydrogenase, and the count of *fdhA* was much higher than that of the other formate dehydrogenase subunits. *fdhA* encoded formate dehydrogenase (coenzyme F_420_) [EC:1.17.98.3 1.8.98.6] which was one of oxidoreductases acting on CH or CH_2_ groups. This demonstrated the oxidization and conversion capabilities into CO_2_. Additionally, higher counts of *cdhA* and *cdhE*/*acsC* were obtained. They both encoded anaerobic carbon-monoxide dehydrogenase (CODH/ACS complex subunit alpha [EC:1.2.7.4]) and acetyl-CoA decarbonylase/synthase (CODH/ACS complex subunit gamma [EC:2.1.1.245]), respectively, and participated in the acetyl-CoA pathway (M00422). Therefore, the CODH/ACS complex gene modules were upregulated, indicating great potential for C1 carbon flux conversion and acetyl-CoA-related metabolism [28,29,30].

Therefore, during anaerobic DMF degradation, the one-carbon intermediates derived from DMF were not exclusively used in methanogenesis. Instead, they might be partitioned in parallel between methylotrophic methanogenesis and further oxidation/integration of C1 intermediates, resulting in the complete mineralization of DMF.

#### 3.3.3. Functional Genes of Nitrogen Flow Pathways

**Figure 6:**
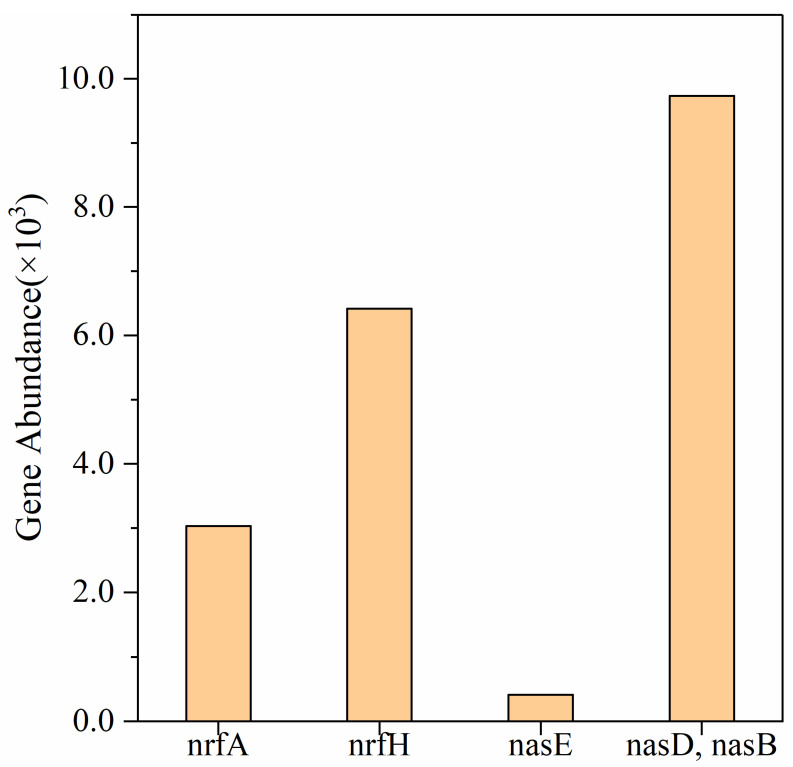
Abundance of nitrogen transformation-related genes.

The nitrogen transformation genes *nrfA*, *nrfH*, *nasE*, and *nasD*/*B* were detected in this system (Figure 6). Among these, *nasD*/*B* showed the highest count of 9738, followed by the count of *nrfH* (6414). *nrfH*/*A* encoded cytochrome c-type nitrite reductase that participated in the nitrogen metabolism/nitrogen cycle system (M00530, dissimilatory nitrate reduction, nitrate ⇒ ammonia). Therefore, the dissimilatory nitrate reduction to ammonium (DNRA) happened in the anaerobic system, whereas *nasD*/*B* encoded nitrite reductase [NAD(P)H] large subunit [EC:1.7.1.4] and participated in the nitrogen cycle pathway of M00531 (assimilatory nitrate reduction, nitrate ⇒ ammonia). NAD(P)H-dependent assimilatory nitrite reduction systems convert inorganic nitrogen into reduced nitrogen forms usable by cells [31]. Therefore, the direction of nitrogen transformation in this system could prefer the reduction of NO_2_^−^ to NH_4_^+^ and subsequent reuse, rather than nitrogen removal via complete denitrification. Moreover, DMF degradation generated nitrogen-containing intermediates and ammonia, indicating that the nitrogen released from DMF in this system tended to enter a reductive nitrogen cycle characterized by nitrogen retention [15,31,32,33].

#### 3.3.4. Methanogenesis Proof: Electron Transfer and Energy Conservation

**Figure 7:**
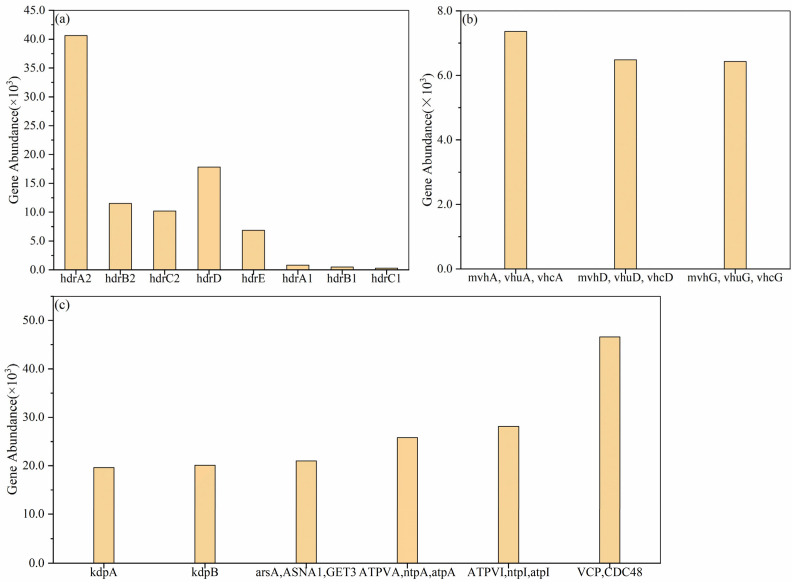
Abundance of methanogenesis-related genes. (**a**,**b**) genes involved in methylamine/dimethylamine/trimethylamine ⇒ methane pathway (M00563); (**c**) genes related to system stability.

The gene *hdrA2* encoding heterodisulfide reductase obtained the higher count of 40,628 and was involved in the methanogenesis of methylamine/dimethylamine/trimethylamine ⇒ methane (M00563), as were *hdrB2*/*C2*/*hdrD*/*E* (Figure 7a). Genes *mvhA* (count 7362) and *mvhG* (count 6432) (homologous to *vhu*/*vhc*) encoded F420-non-reducing hydrogenase and participated in the methylamine/dimethylamine/trimethylamine ⇒ methane pathway (M00563). *mvhD* (homologous to *vhu*/*vhc*) with a count of 6484 participated in the methanol ⇒ methane pathway (M00563) (Figure 7b). This signified a strong capacity for sulfide reduction and hydrogen-dependent electron bifurcation. Previous studies have reported that the *Hdr* complex was responsible for reducing CoM-S-S-CoB during methanogenesis. In contrast, the *Mvh* complex cooperated with *Hdr* for electron bifurcation and provided reducing power for the terminal step of methanogenesis [34,35,36]. Additionally, the system contained *ATPVA*/*ATPVI* (*ntpA*/*ntpI*/*atpA*/*atpI*) encoding V/A-type H+/Na+-transporting ATPase. These were involved in the pathway of oxidative phosphorylation (M00159) (Figure 7c) with counts of 25,812 and 28,132, respectively. Therefore, archaea possessed strong potential for membrane-associated energy conservation and ATP synthesis, thus providing an energy basis for sustained methane metabolism under low-energy-yield conditions [36]. Therefore, in the present study, methanation was supported by the substrate conversion capabilities and relatively complete electron transfer and energy conversion systems.

#### 3.3.5. Denitrification and Anaerobic Ammonium Oxidation

High levels of *kdpA*, *kdpB*, *arsA/*ASNA1*/GET3*, and *VCP*/*CDC48* were also found in the anaerobic system (Figure 7c). Among them was *kdpA*/*B*, a high-affinity potassium uptake system that is typically associated with osmoregulation and maintenance of ion homeostasis. *VCP*/*CDC48*, with the highest count of 46,576, encoding transitional endoplasmic reticulum ATPase, belongs to the AAA+ ATPase family and was significantly associated with protein homeostasis, protein complex remodeling, and stress responses [37,38,39]. DMF, a polar organic solvent, could cause membrane and metabolic stress in cells. Therefore, the high abundance of those genes indicated strong capabilities of environmental adaptation and functional maintenance of microorganisms, particularly the archaeal population, which facilitated stable metabolic output under high-load DMF conditions.

### 3.4. Effects of Environmental Factors on Microbial Community

#### 3.4.1. Effects on Microbial Community Diversity

**Figure 8:**
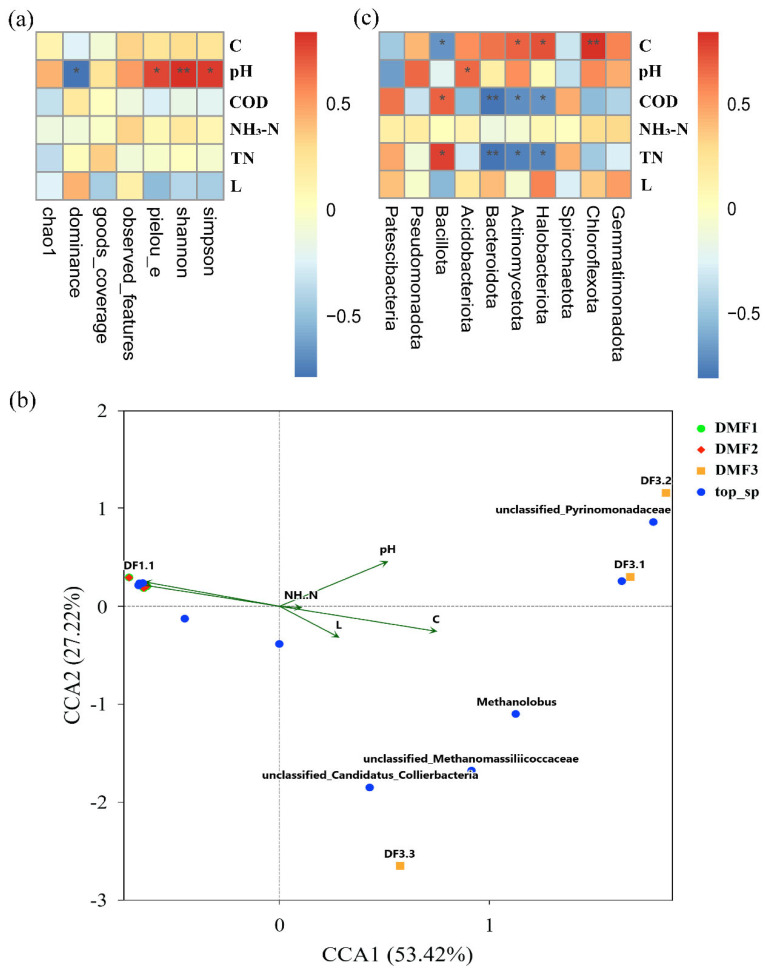
Analysis between microbial community and physicochemical properties of DMF wastewater. (**a**) Correlation relationship between microbial diversity and physicochemical properties; (**b**) redundancy analysis (RDA) and (**c**) correlation between phylum level and physicochemical properties. COD, chemical oxygen demand; TN, total nitrogen; DMF, N,N-dimethylformamide; C, DMF concentration; L, gas production; NH_3_-N, ammonium nitrogen. (* means *p* < 0.05; ** means *p* < 0.01.

The correlation relationship between the physicochemical properties of DMF wastewater at different DMF concentrations and the microbial diversity indices of the anaerobic sludge was analyzed (Figure 8a). The main physicochemical parameters included COD, TN, DMF concentration (C), gas production (L), and NH_3_-N. It was indicated that pH had the greatest impact on microbial diversity. It was significantly negatively correlated with the dominance index (*p* < 0.05) and significantly positively correlated with the Shannon and Simpson indices (*p* < 0.05) and the Pielou_e index (*p* < 0.01). It was found that the growth of DMF-degrading bacteria (DMF-3) was inhibited when the initial pH was 8.0–9.0, the strain grew faster at pH = 7.0, and the favorable initial pH for the growth of DMF-3 was 6.0–7.0 [33]. C, NH_3_-N, and TN were positively correlated with diversity indices, while COD and L showed an opposite correlation. It has been reported that the Shannon index was negatively correlated with inhibition luminosity of sludge toxicity [18]. Therefore, as COD increased, sludge toxicity increased, resulting in a decrease in microbial diversity.

#### 3.4.2. Effects on Microbial Community Composition

RDA was used to analyze the relationship between the environmental factors and microbial community composition (Figure 8b). The two axes, CCA1 and CCA2, explained 64.85% and 23.3% of the variance, respectively, accounting for 88.15% of the total variance. It effectively reflected the correlation between wastewater physicochemical properties and the structure of the anaerobic sludge microbial community. The distribution of the environmental factors was relatively dispersed. L was positively correlated with pH, C, and NH_3_-N, but negatively correlated with TN and COD. This is similar to the DMF-degrading performance results described in Section 3.1. Furthermore, the positive correlations between pH, C, and NH_3_-N, TN and COD suggested potential synergistic effects of these environmental factors on the microbial community. Furthermore, they influenced the degradation efficiency of DMF and its metabolic pathways, particularly with respect to pH, COD, TN, and C. Therefore, they could be set as indicators for assessing the system stability and process performance. Moreover, pH, C, and NH_3_-N were positively correlated with Halobacteriota, Actinomycetota, Pseudomonadota, and Thermoolasmatota. The same relationship was found between COD and TN, and between Bacillota and Spirochaetota. The differences indicated different responses of the microbial community to the DMF influent properties.

The correlation relationships between the dominant microbial phyla and environmental factors at different DMF concentrations showed that environmental factors significantly affected the distribution of microorganisms at the phylum level (Figure 8c). C showed a significant positive correlation with the phyla Chloroflexota, Halobacteriota, Actinomycetota, and Bacteroidota (*p* < 0.05), and a significant negative correlation with Bacillota (*p* < 0.05). pH showed a significant positive correlation with Gemmatimonadota and Pseudomonadota (*p* < 0.05) and a significant negative correlation with Patescibacteria (*p* < 0.05). The COD showed a significant positive correlation with Bacillota and a significant negative correlation with Chloroflexota, Halobacteriota, and Bacteroidota (*p* < 0.05). TN showed a significant positive correlation with Bacillota and a significant negative correlation with Halobacteriota and Bacteroidota (*p* < 0.05). However, no significant correlation was found between L and microbial phyla (correlated but not significant, *p* > 0.05). Therefore, the DMF concentration and COD were parameters that influenced the process parameters (pH, TN, and NH_3_-N), which, in turn, influenced microbial composition and function, ultimately manifesting as differences in DMF metabolic pathways and gas production.

## 4. Discussion

### 4.1. Effects of DMF Concentration on DMF Removal Efficiency

Results showed that at a DMF concentration range of 100–2000 mg/L, ORL increased from 250 to 9000 mg/L, and the removal rates of DMF and COD remained stable over 90% and 80%, respectively. This is consistent with the previous study, in which mixed DMF-degrading activated sludge and anaerobic granular sludge were used, and over 96% DMF removal efficiency and a high methane production rate were obtained in continuous operation of UASB, whereas OLR (1.63–4.22 g COD L^−1^ d^−1^) [14] was much lower than this study (0.25–9.0 g COD L^−1^ d^−1^). Also, when 200–400 mg/L DMF was treated using an aeration tank, OLR was only 0.2–0.4 g COD L^−1^ d^−1^ [40], while it was less than 0.5 g COD L^−1^ d^−1^ using an eco-tank [41]. Therefore, the co-culture of anaerobic sludge could achieve higher DMF degradation and methane conversion. However, when DMF concentration increased to 3500 mg/L, the degrading efficiency decreased. The main reasons were (1) as DMF concentration increased, the sludge toxicity increased, and an increase of 25 to 45% resulted when DMF concentration ranged from 40 to 200 mg/L [18], and (2) the total sludge toxicity was primarily contributed by the degradation of DMF [18]. DMA and MMA were the key intermediates during the DMF-degrading process. It could be found that both of them increased as DMF load increased (<2000 mg/L) without accumulation owing to the methane production. At 3500 mg/L, DMA and MMA increased obviously, and thus toxicity increased.

### 4.2. Changes in Microbial Composition Under Different DMF Loads

As the DMF concentration increased, the relative abundances of Pseudomonadota, Actinomycetota, Bacteroidota, Acidobacteriota, Halobacteriota, and Thermoplasmatota increased, whereas those of Patescibacteria and Bacillota decreased. This indicated that high concentrations of DMF exerted significant directional selection pressure on community structure. In the anaerobic system, DMF acted as both a substrate and an organic solvent stressor; thus, it favored the enrichment of functional microbial groups that tolerated DMF toxicity and could participate in its transformation and utilization as intermediates. Previous studies have shown that DMF degradation potential was primarily distributed within Pseudomonadota and Actinomycetota, suggesting that these groups carried out the initial transformation and/or key metabolic steps of DMF. Organic solvent tolerance mechanisms, such as efflux pumps, were commonly found in Pseudomonadota. These contributed to their competitive advantage under high-DMF conditions [21,27]. Bacteroidota generally degrades complex organic matter. In the present study, they utilized extracellular polymeric substances, cell lysis products, and other secondary organic substrates to further enhance the DMF degrading efficiency. The increase in Acidobacteriota might be related to their strong environmental adaptability, stress tolerance, and responses to complex niche changes, especially at high DMF loads (>2000 mg/L) [42,43].

Anaerobic DMF degradation typically depended on the syntrophic metabolism between bacteria and methanogenic archaea. DMF could be converted into intermediates such as DMA and formate, which then served as substrates for methylotrophic or other methanogenic archaea. Therefore, increases in Halobacteriota and Thermoplasmatota suggested enhanced utilization of methylated intermediates and terminal methanogenesis in the system [15,44,45]. In contrast, the significant decrease in Patescibacteria was due to their generally ultra-small cell size, reduced genome, limited metabolic and biosynthetic capabilities, and hosts or symbionts that depended on survival habits. Under conditions of strong stress and substrate specificity induced by high DMF concentrations, this type of dependent group typically showed lower competitiveness with functional bacteria in terms of dependent tolerance and metabolic capability, resulting in a decrease in relative abundance [46,47,48]. Furthermore, although Bacillota were generally considered the most important hydrolytic and acidogenic bacteria in anaerobic digestion, their ecological advantages were more dependent on the primary digestion process of conventional complex organic matter, such as carbohydrates and proteins. When the carbon flow gradually shifted from the general hydrolysis/acidification of organic matter to the specific transformation and serial utilization of methylated intermediates of DMF, its relative contribution was replaced by Pseudomonadota, Actinomycetota, and functional archaea [48,49]. Although some Bacillota species can enhance their survival rate under adverse conditions by forming spores, sporulation is a survival strategy rather than active growth. Therefore, the decrease in their relative abundance did not necessarily imply complete inactivation but rather reflected their transition from active metabolism to a low-activity or dormant state under a high DMF load [50,51]. Overall, the results of this study indicated that the community changes in the anaerobic system under high DMF concentration exhibited a trend of restructuring from a “dependent/general fermentative” population to a “tolerant–specific transforming–syntrophic methanogenic” population. It provided an important ecological basis for maintaining the stable operation of high-load DMF anaerobic degradation.

### 4.3. Key Functional Genes Involved in DMF Metabolic Mechanisms

This study demonstrated that DMF metabolism in the co-cultured anaerobic system did not follow a single pathway but exhibited a clear pattern of “carbon flux splitting and nitrogen flux redistribution.” From the carbon flux perspective, the relatively high abundance of *mcr*, *mta*, *mtb*, *mtt*, and *mtm* genes indicated the potential for complete methylotrophic methanogenesis. The higher abundance of *mtb* and *mtt* compared to *mta* and *mtm* suggested that DMA/TMA was the primary methyl donor in methanogenesis [15,21,27]. This is consistent with previous studies, which showed that anaerobic DMF degradation produced intermediates such as DMA and formate as substrates for methanogenic pathways [15,21]. Simultaneously, the high abundances of *fdh* and *cdh*/*acs* indicated that only a portion of the one-carbon intermediates generated from DMF degradation entered methanogenesis. At the same time, many of them could participate in CO_2_ formation via formate oxidation and CODH/ACS-mediated C1 transformation or acetyl-CoA-related carbon flux integration pathways [28,29,30]. Therefore, the more reasonable explanation for the DMF carbon flux in this study was a parallel pathway in which some carbon proceeded methylotrophic methanogenesis driven by methylamine, and the rest entered CO_2_ production or the central carbon metabolic network via C1 oxidation/integration.

From the nitrogen flux perspective, the combination of *nrfA*/*H* and *nasE*/*D (nasB)* genes supported the occurrence of DNRA and assimilatory nitrite reduction, which aligned with the presence of *Desulfovibrio* in anaerobic sludge. However, it did not support complete denitrification for nitrogen removal [31]. This meant that nitrogen-containing components released during DMF degradation were more likely to be converted to NH_4_^+^ and retained or reused within the system, rather than ultimately discharged as N_2_ [31,32,33]. From an ecological perspective, this nitrogen-retention transformation pattern favored maintaining a nitrogen supply for microorganisms under anaerobic conditions. Also, it indicated that DMF degradation involved not only carbon metabolism restructuring, but also a shift in the internal nitrogen cycling mode.

Notably, the high abundance of the *Hdr-Mvh* electron transfer module, A/V-type ATPases, and stress adaptation modules, such as *Kdp*/*VCP*, provided crucial support for the stable operation of the aforementioned metabolic pathways. The synergistic effects of *Hdr* and *Mvh* suggested that methanogenesis was carried out not only by substrate supply but also by a complete reducing power transfer and energy coupling mechanism [34,35,36]. Enrichment of the Kdp system, *VCP*/*CDC48*, and related ATPases indicated that the microbial community also had strong capabilities to maintain ion homeostasis, regulate protein homeostasis, and adapt to environmental stress [37,38,39]. Therefore, the stable operation of the system under high DMF load did not depend solely on the enhancement of a single metabolic pathway. It was achieved by a composite functional network comprising “primary metabolic pathways + energy support + homeostasis maintenance.”

## 5. Conclusions

The co-cultured anaerobic system exhibited improved DMF degradation efficiency. When the DMF concentration ranged from 100 mg/L to 2000 mg/L, the COD concentration increased, and the removal rates of COD and DMF from the effluent wastewater remained above 80% and 90%, respectively. Process stability and nitrogen cycling improved. DMF degraded via the metabolic pathway of DMF → DMA → MMA → CH_4_/CO_2_. It represented a mature, efficient, highly functionally differentiated, and self-regulating anaerobic ecosystem. The microbial community reconstructed as DMF concentration increased. Enrichment of tolerance and functionality groups, specifically, Pseudomonadota, Actinomycetota, and Bacteroidota, was observed. The dominant genera in the methanogenic metabolic pathway shifted from *Methanothrix* to *Methanolobus* and *unclassified_Methanomassiliicoccaceae*. *Desulfovibrio*, *Pseudomonas*, and *Hyphomicrobium* were the key hydrolytic bacteria in DMF. Metagenomic results showed that DMF metabolism exhibited distinct multi-pathway splitting characteristics. Carbon flux was achieved by the synergistic transformation of methylamine-driven methanogenesis and the further oxidation/integration of one-carbon intermediates. Nitrogen flux tended to enter a reductive nitrogen cycle characterized by preservation and reuse.

## Figures and Tables

**Table 1:** Alpha diversity analysis of anaerobic microbial community.

Sample	Coverage	Chao1	Shannon	Simpson
DMF1	0.998 ± 0.002	753.538 ± 139.349	6.552 ± 0.379	0.955 ± 0.022
DMF2	0.997 ± 0.003	763.226 ± 151.456	6.552 ± 0.379	0.955 ± 0.022
DMF3	0.998 ± 0.001	1291.654 ± 615.443	8.065 ± 2.009	0.970 ± 0.036
*p*	-	0.213	0.278	0.760

**Table 2:** The genera related to DMF degradation in anaerobic sludge.

No.	Genus Name	Relative Abundance (%)		
		DMF1	DMF2	DMF3
1	*Desulfovibrio*	0.825 ± 0.127	0.827 ± 0.129	0.568 ± 0.468
2	*Bacteroides*	0.183 ± 0.014	0.177 ± 0.004	0.467 ± 0.454
3	*Aeromonas*	0.430 ± 0.279	0.427 ± 0.282	0.009 ± 0.013
4	*Zavarzinia*	0.224 ± 0.116	0.220 ± 0.009	0
5	*Pseudoalteromonas*	0.525 ± 0.295	0.528 ± 0.297	0
6	*Hyphomicrobium*	0	0	0.058 ± 0.090
7	*Methanolobus*	0	0	8.100 ± 4.402
8	*Methanothrix*	1.068 ± 0.750	1.062 ± 0.750	0.247 ± 0.345
9	*Methanomassiliicoccus*	0.002 ± 0.002	0.001 ± 0.002	0.772 ± 0.517
10	*unclassified_Methanomassiliicoccaceae*	0.002 ± 0.004	0.002 ± 0.004	5.185 ± 5.122
11	*unclassified_Bacteroidales_RF16_group*	0	0	0.078 ± 0.135
12	*unclassified_Bacteroidetes_vadinHA17*	0.110 ± 0.038	0.110 ± 0.021	0.110 ± 0.064
13	*Clostridium*	2.574 ± 0.476	2.556 ± 0.468	1.047 ± 1.140
14	*Trichococcus*	3.900 ± 1.207	3.913 ± 1.214	0.033 ± 0.029

## Data Availability

The original contributions presented in this study are included in the article/Supplementary Materials. Further inquiries can be directed to the corresponding authors.

## Supplementary Materials

The following supporting information can be downloaded at: https://www.mdpi.com/article/10.3390/microorganisms14061172/s1, Figure S1: Rarefaction curves of microbial communities in anaerobic sludge under different DMF concentrations. DF1.1–DF1.3, three repetitions of DMF concentration 100 mg/L; DF2.1–DF2.3, three repetitions of DMF concentration 2000 mg/L; DF3.1–DF3.3, three repetitions of DMF concentration 3500 mg/L.

## Abbreviations

DMF, N,N-dimethylformamide; UASB, up-flow anaerobic sludge blanket; HRT, hydraulic retention time; TMA, trimethylamine; DMA, dimethylamine; MMA, monomethylamine; HCOOH, formic acid; NMF, N-methylformamide; HCHO, formaldehyde; FA, formamide; AD, anaerobic digestion; COD, chemical oxygen demand; NH_3_-N, ammonia nitrogen; TN, total nitrogen; L, gas production; C, concentration of DMF; RDA, redundancy analysis; VBPR, volumetric biogas production rate; VLR, volumetric loading rate; ASVs, Amplicon Sequence Variants; MCR, methyl-coenzyme M reductase; FDH, formate dehydrogenase; DNRA, dissimilatory nitrate reduction to ammonium.
