# Supplementary material for: Microbial Responses and Metabolic Mechanisms During Anaerobic Degradation of N,N-Dimethylformamide by Co-Cultured Sludge
Source: Microorganisms. 2026 May 22;14(6):1172. doi: 10.3390/microorganisms14061172 (PMC13304083; doi:10.3390/microorganisms14061172)
Supplement: Supplementary file 1 [file microorganisms-14-01172-s001.zip › microorganisms-4261621-supplementary.pdf]

*Supplementary Materials*

# Microbial Responses and Metabolic Mechanisms During Anaerobic Degradation of N,N-Dimethylformamide by Co-Cultured Sludge

**Jianrong Liu [1], Yingying Song [2], Hongruo Ma [1], Chunlan Mao [3,*] and Zuoyan Chen [1,*]**

1 Gansu Natural Energy Institute, Lanzhou 730000, China
2 Gansu Analysis and Research Center, Lanzhou 730000, China
3 State Key Laboratory of Ecological Safety and Sustainable Development in Arid Lands, Lanzhou Eco-Agriculture Experimental Research Station, Northwest Institute of Eco-Environment and Resources, Chinese Academy of Sciences, Lanzhou 730000, China
* Correspondence: maochunlan@nieer.ac.cn (C.M.); czy805@gmail.com (Z.C.)

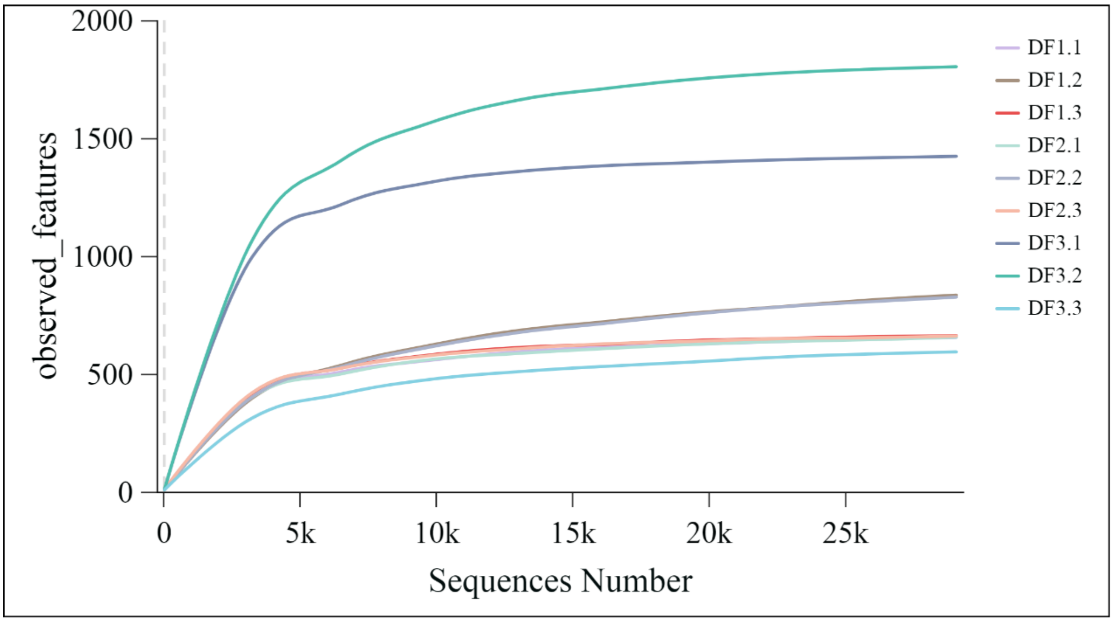


**Figure S1.** Rarefaction curves of microbial communities in anaerobic sludge under different DMF concentrations. DF1.1–DF1.3, three repetitions of DMF concentration 100 mg/L; DF2.1–DF2.3, three repetitions of DMF concentration 2000 mg/L; DF3.1–DF3.3, three repetitions of DMF concentration 3500 mg/L.
